# Donor genetic burden for cerebrovascular risk and kidney transplant outcome

**DOI:** 10.1007/s40620-024-01973-0

**Published:** 2024-05-29

**Authors:** Kane E. Collins, Edmund Gilbert, Vincent Mauduit, Katherine A. Benson, Elhussein A. E. Elhassan, Conall O’Seaghdha, Claire Hill, Amy Jayne McKnight, Alexander P. Maxwell, Peter J. van der Most, Martin H. de Borst, Weihua Guan, Pamala A. Jacobson, Ajay K. Israni, Brendan J. Keating, Graham M. Lord, Salla Markkinen, Ilkka Helanterä, Kati Hyvärinen, Jukka Partanen, Stephen F. Madden, Sophie Limou, Gianpiero L. Cavalleri, Peter J. Conlon

**Affiliations:** 1 School of Pharmacy and Biomolecular Sciences, Royal College of Surgeons in Ireland, Dublin, Ireland; 2 The Science Foundation Ireland FutureNeuro Centre of Excellence, Dublin, Ireland; 3 SFI Centre for Research Training in Genomics Data Science, University of Galway, Galway, Ireland; 4 Center for Research in Transplantation and Translational Immunology, UMR1064, Nantes University, Ecole Centrale Nantes, INSERM, Nantes, France; 5 Department of Nephrology and Transplantation, Beaumont Hospital, Dublin, Ireland; 6 Department of Medicine, Royal College of Surgeons in Ireland, Dublin, Ireland; 7 Centre for Public Health, Queen’s University Belfast, Belfast, United Kingdom; 8 Department of Epidemiology, University of Groningen, University Medical Center Groningen, Groningen, The Netherlands; 9 Department of Internal Medicine, Divison of Nephrology, University of Groningen, University Medical Center Groningen, Groningen, The Netherlands; 10 Division of Biostatistics, School of Public Health, University of Minnesota, Minneapolis, Minnesota USA; 11 College of Pharmacy, University of Minnesota, Minneapolis, Minnesota USA; 12 Department of Surgery, Perelman School of Medicine, University of Pennsylvania, Philadelphia, Pennsylvania USA; 13 Faculty of Biology, Medicine and Health, University of Manchester, Manchester, United Kingdom; 14 Finnish Red Cross Blood Service, Research and Development, Biomedicum 1, Helsinki, Finland; 15 Transplantation and Liver Surgery, Helsinki University Hospital, Helsinki, Finland; 16 Data Science Centre, Royal College of Surgeons in Ireland, Beaux Lane House, Dublin, Ireland

**Keywords:** Donors, Genetics, Polygenic risk scores, Post-transplant eGFR, Stroke

## Abstract

**Background and hypothesis:**

Kidney grafts from donors who died of stroke and related traits have worse outcomes relative to grafts from both living donors and those who died of other causes. We hypothesise that deceased donors, particularly those who died of stroke, have elevated polygenic burden for cerebrovascular traits. We further hypothesise that this donor polygenic burden is associated with inferior graft outcomes in the recipient.

**Methods:**

Using a dataset of 6666 deceased and living kidney donors from seven different European ancestry transplant cohorts, we investigated the role of polygenic burden for cerebrovascular traits (hypertension, stroke, and intracranial aneurysm (IA)) on donor age of death and recipient graft outcomes.

**Results:**

We found that kidney donors who died of stroke had elevated intracranial aneurysm and hypertension polygenic risk scores, compared to healthy controls and living donors. This burden was associated with age of death among donors who died of stroke. Increased donor polygenic risk for hypertension was associated with reduced long term graft survival (HR: 1.44, 95% CI [1.07, 1.93]) and increased burden for hypertension, and intracranial aneurysm was associated with reduced recipient estimated glomerular filtration rate (eGFR) at 1 year.

**Conclusions:**

Collectively, the results presented here demonstrate the impact of inherited factors associated with donors' death on long-term graft function.

**Graphical Abstract:**

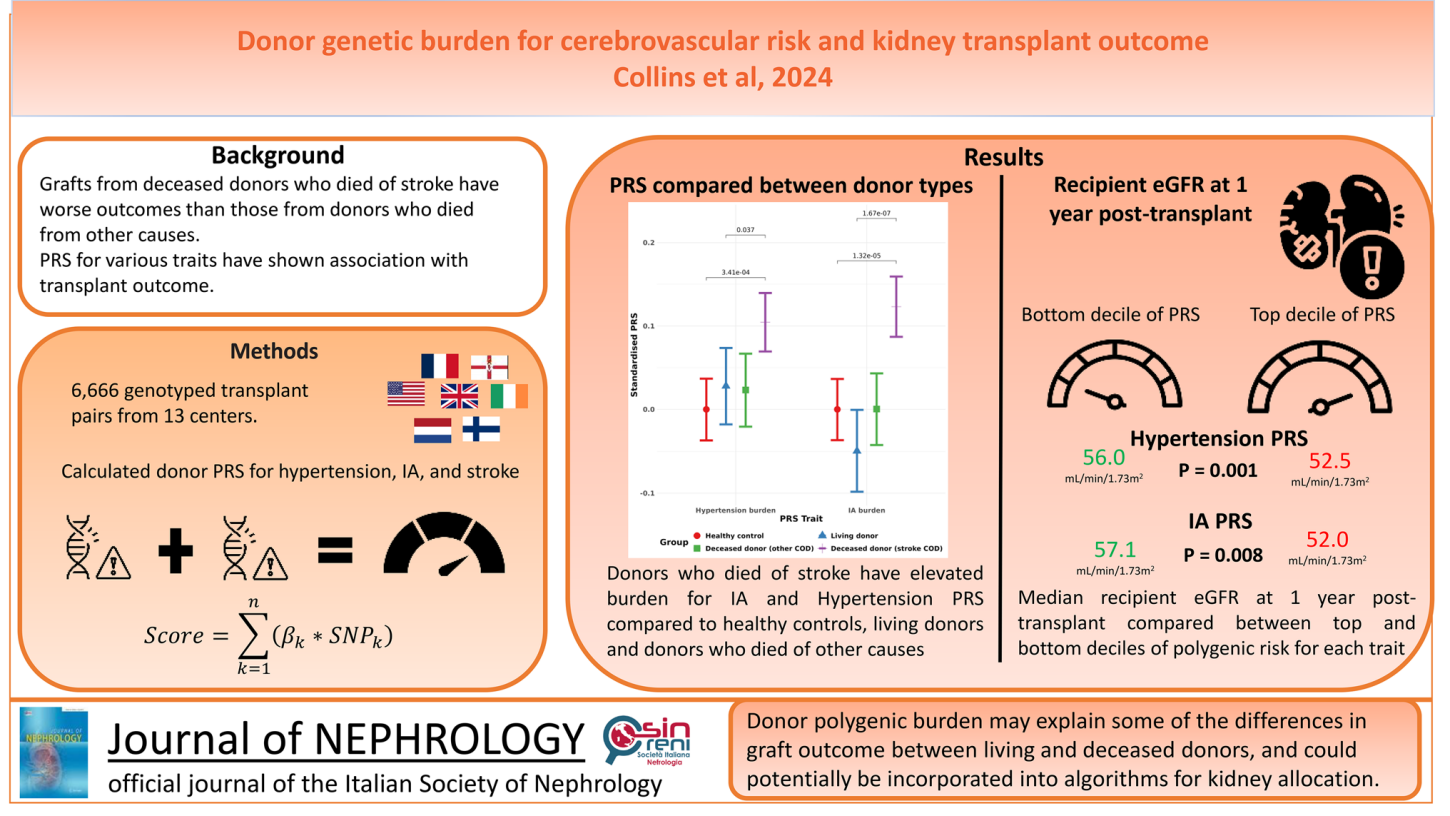

**Supplementary Information:**

The online version contains supplementary material available at 10.1007/s40620-024-01973-0.

## Introduction

Kidney transplant outcomes are influenced by factors including donor age and sex, whether the donor is living or deceased, clinical era of transplant, donor cause of death, and HLA mismatch [1, 2]. It is well established that organs from living donors generally have superior outcomes compared to those from deceased donors [3]. Further, grafts from deceased donors who died of spontaneous intracranial haemorrhage or stroke have worse outcomes than those from donors who died of other causes, such as trauma [4, 5]. Understanding the factors underlying the poor performance associated with stroke organs could help improve transplant outcomes.

Hypertension is the most prevalent clinical risk factor for stroke and intracranial aneurysm (IA) [6]. Hypertension, intracranial aneurysm and stroke all are highly genetic and have undergone genome-wide association studies (GWAS) [7–9], which have identified multiple risk loci, each study explaining up to 22% of SNP-based heritability.

Polygenic Risk Scores (PRSs) can be leveraged to quantify individual genetic burden for a trait using summary statistics from genome-wide association studies. Polygenic risk scores estimate the cumulative effect of common genetic variation on an individual’s disease status weighted by estimated effect size [10, 11]. In the case of ischaemic stroke (IS), previous work has demonstrated that when combined with clinical risk factors, an ischaemic stroke polygenic risk score has the potential to significantly improve risk classification [12]. In addition, intracranial aneurysm polygenic risk score has been shown to predict aneurysmal subarachnoid haemorrhage [13].

Previous studies have demonstrated the impact of both donor and recipient polygenic risk score on transplant outcome. For example, donor and recipient polygenic burden for estimated Glomerular Filtration Rate (eGFR) has been correlated with transplant outcome. Recipient burden for skin cancer has also been associated with skin cancer post-transplant [14, 15]. Other studies [16, 17] have shown an association between donor genetic risk scores in interleukin-6 and biopsy-proven rejection.

Given the poor transplant outcome associated with deceased and stroke-related organs in particular, here we investigate the influence of donor polygenic burden for stroke, intracranial aneurysm, and hypertension on donor age of death and kidney transplant outcome. We hypothesized that the polygenic burden of kidney donors is significantly different between living and deceased donors, and between deceased donors who died from stroke and from other causes. We further hypothesized that differences in donor burden would manifest in different transplant outcomes as measured by eGFR and graft survival.

## Methods

### Ethics statement

The study was approved by the relevant ethics committees at each site (Data Characteristics section of Supplementary Materials).

### Study design

We hypothesized that the polygenic burden of kidney donors is significantly different between living and deceased donors, and between deceased donors who died from stroke and from other causes. To test this, we calculated the polygenic risk score for hypertension, intracranial aneurysm, and stroke across 6666 kidney donors and 2870 healthy controls from seven cohorts of European ancestry. Polygenic risk score was compared between healthy controls, living donors, donors who died of stroke, and donors who died of other causes. We investigated the impact of these polygenic risk scores on donor age of death, meta-analysing across all the 7 cohorts. To test the hypothesis that differences in donor burden would manifest in different transplant outcomes, we investigated the impact of polygenic risk score on graft outcomes through a meta-analysis across the same seven cohorts.

### Cohort characteristics

We assembled seven European ancestry cohorts of paired kidney transplant donors and recipients. Each cohort had phenotype data pertaining to donor type, donor cause of death, and transplant outcomes, including eGFR at 1- and 5-years post-transplant. These cohorts included 3 types of donors: (1) living donors, (2) deceased donors that died of stroke and intracranial haemorrhage (henceforth referred to as donors that died of stroke), and (3) deceased donors that died of other causes (predominantly cerebral trauma but also asphyxia). As kidneys from paediatric donors are not fully developed, such kidneys might have lower graft function, and would thus not follow the well-established linear relationship between increasing donor age and decreasing graft function. Such donors were thus excluded from the analysis. Please see Data Characteristics in the Supplementary Materials for a full description of the cohorts.

All seven cohorts had genotype array data available which was subject to standard quality control for minor allele frequency, genotyping rate and missingness. All individuals were selected to be unrelated to the level of 3rd degree using KING [18] software, as well as of European ancestry, identified using principal components of ancestry. See Data QC and Procession section of Supplementary Materials for further details.

Supplementary Table 1 provides similar cohort characteristics broken down by donor type (living vs deceased). eGFR was calculated using the CKD-EPI Creatinine Equation (2021) [19]. Individuals (*n* = 2870) from the Peoples of the British Isles dataset were used as controls for all cohorts [20].

### Polygenic risk score calculation

We calculated donor polygenic risk score for stroke, intracranial aneurysm and hypertension using published genome-wide association studies of European ancestry for each trait [7–9]. Further details of these genome-wide association studies can be found in Supplementary Table S2. Polygenic risk scores were calculated using PRSice2 [21], selecting alleles with a *p*-value threshold greater than 0.5, physical distance threshold for clumping of 250 kb and linkage disequilibrium threshold (*r*^*2*^) of 0.1. Analysis was conducted in R, using version 4.2.1 (2022–06–23) [22].

### Comparison of polygenic burden between groups

We used a Kruskal–Wallis test, and a Dunn test to investigate differences in polygenic burden between our 4 groups of individuals (healthy controls, living donors, deceased donors who died of stroke, and deceased donors who died of other causes). We conducted these tests separately for each of the three polygenic risk scores. The Dunn test accounts for multiple testing.

### Influence of donor polygenic burden on age of death in donors who died of stroke

We created meta-regressions using the R package *meta* to investigate the role of polygenic burden for each trait on age of death in donors who died of stroke. Separate models were constructed for each polygenic risk score, taking donor sex and the first four principal components of genetic ancestry as covariates. All the assumptions of a linear model were checked (residuals vs fitted, normal Q-Q, scale-location, and residuals vs leverage). The variance explained by each polygenic risk score was calculated along with the heterogeneity of each model.

Donors who died of stroke were split into 3 groups for each polygenic risk score, defined in the following way: high risk (Top 10% of risk for each polygenic risk score), intermediate risk (Middle 80% of risk), and low risk (Bottom 10% of risk). This was done separately for each polygenic risk score. Median donor age of death was compared between each of these groups.

### Impact of donor polygenic risk score on graft survival

All donors were split into the same 3 groups of risk (high, intermediate and low, as previously defined) for each trait. Median graft survival was compared between each of these groups.

We created meta-regressions using the R package *meta* to investigate the role of polygenic burden for each trait on graft survival. Separate Cox proportional hazards models were constructed for each polygenic risk score, each taking donor and recipient sex, donor and recipient age, year of transplant, and whether it was the recipient’s first transplant or not, and the first four principal components of genetic ancestry as covariates. Donor age initially failed the proportional hazards assumption, so was then stratified into 2 categories (< 50 years, ≥ 50 years). The variance explained by each polygenic risk score was calculated along with the heterogeneity of each model. Additionally, this analysis was split by donor type (stroke vs other cause of death (COD)) to investigate if there was a difference in the impact of polygenic risk score on graft survival between the two groups.

### Impact of donor polygenic risk score on graft function

Recipient graft function was defined as eGFR at 1- and 5- years post-transplant. We created 3 meta-regression models (one for each polygenic risk score) using the R package *meta* to predict recipient eGFR at 1-year post-transplant. These models all included the following covariates: donor sex, donor age, donor type, recipient age, recipient sex, year of transplant, and first transplant. All the standard assumptions of linear models were tested. The variance explained by each polygenic risk score was calculated along with the heterogeneity of each model. A similar process was carried out to predict eGFR at 5 years.

We also created a similar model to predict eGFR at 5 years. We tested for the assumptions of non-linearity, homogeneity of variance, influential observations, collinearity, normality of residuals, and normality of random effects (Supplementary Figures S4, S5). The heterogeneity of the models was also tested using the *rma* function from the *metafor* R package.

Again, we split all the individuals into the same 3 groups of risk (high, intermediate and low, as previously defined) for each trait, and compared eGFR at 1 and 5 years between these groups, as well as graft survival.

## Results

Data from 6666 individuals passed QC and were included in the analysis. Clinical characteristics of the dataset are shown in Table 1. The mean donor age was 49 years, with more male donors (53%) than female. Among the donors, 1582 (24%) were living, 3113 (47%) died of stroke and, 1971 (30%) died of other causes of death. Those who died of stroke were the oldest (54 years old), followed by living donors (45 years old) and donors who died of other causes (44 years old) (see Supplementary Table S1).

**Table 1:** Summary statistics for the meta-analysis split into each cohort

Variable	Overall	DeKAF	FRCBS	GEN03	KiT-GENIE	QUB	TL	UKIRTC
Number of patients	6,666	684	929	476	1853	133	696	1895
Donor age, median (min–max)	50 (18–90)	44 (18–70)	58 (18–77)	45 (18–71)	55 (18–90)	40 (18–68)	46 (18–72)	47 (18–81)
Female donor, *n* (%)	3106 (47)	405 (59)	433 (47)	273 (57)	776 (42)	58 (44)	344 (49)	817 (43)
Donor type								
Living, *n* (%)	1582 (24)	684 (100)	0 (0)	476 (100)	319 (17)	0 (0)	103 (15)	0 (0)
Other COD, *n* (%)	1971 (30)	0 (0)	311 (33)	0 (0%)	655 (35)	55 (41)	249 (36)	701 (37)
Stroke COD, *n* (%)	3113 (47)	0 (0)	618 (67)	0 (0)	879 (47)	78 (59)	344 (49)	1194 (63)
Recipient age, median (min–max)	51 (0–84)	51 (0–83)	57 (18–79)	51 (1–81)	53 (18–84)	43 (4–72)	49 (15–74)	47 (18–79)
Female recipient, *n* (%)	2391 (36)	231 (34)	290 (31)	177 (37)	657 (35)	56 (42)	282 (41)	698 (37)
Year of transplant, median (min–max)	2007 (1981–2020)	2008 (2006–2010)	2014 (2007–2017)	2014 (2012–2015)	2011 (1999–2020)	1997 (1986–2005)	2000 (1993–2008)	2001 (1981–2007)
PRD								
Glomerulonephritis	772 (12)	175 (26)	125 (13)	162 (34)	0 (0)	9 (7)	139 (20)	162 (9)
IgA nephropathy	313 (5)	0 (0)	119 (13)	0 (0)	0 (0)	10 (8)	49 (7)	135 (7)
Other	3283 (49)	198 (29)	293 (32)	122 (26)	1569 (85)	74 (56)	311 (45)	716 (38)
PKD	990 (15)	107 (16)	191 (21)	76 (16)	284 (15)	20 (15)	89 (13)	223 (12)
Type 2 diabetes	366 (6)	175 (26)	76 (8)	86 (18)	0 (0)	4 (3.)	5 (1)	20 (1)
Unknown	942 (14)	29 (4.2)	125 (13)	30 (6)	0 (0)	16 (12)	103 (15)	639 (34)
First transplant, *n* (%)	5852 (88)	603 (88)	929 (100)	420 (88)	1450 (78)	133 (100)	637 (92)	1680 (89)
Follow up, median (min–max)	5 (0–25)	2 (0.4–5)	3 (0–10)	2 (0.4–3)	6 (0–21)	8 (0–25)	7 (0–17)	8 (0–25)
Graft status, *n* (%)								
Censored	5577 (84)	671 (98)	871 (94)	474 (99.6)	1439 (78)	92 (69)	586 (84)	1444 (76)
Rejected	1089 (16)	13 (2)	58 (6)	2 (0.4)	414 (22)	41 (31)	110 (16)	451 (24)
eGFR at 1 year, median (min–max)	53 (4–185)	60 (4–178)	54 (6–135)	62 (16–185)	51 (8–129)	NA	47 (4–124)	50 (6–124)
Unknown, *n* (%)	1030 (15)	0 (0)	269 (29)	0 (0)	168 (9)	133 (100)	59 (8)	401 (21)
eGFR at 5 years, median (min–max)	49 (3–124)	NA	50 (13–106)	NA	50 (7–122)	NA	50 (3–124)	48 (6–121)
Unknown	3865	684	829	476	839	133	215	689

Descriptive characteristics for the following cohorts: eGFR is in units of mL/min/1.73 m^2^. First transplant refers to whether it is the recipient’s first transplant or not. The numbers in each cohort refer to the number of donor kidneys rather than just the number of donors, as one deceased donor may donate two kidneys. Follow up is the mean death-censored graft survival time. Further information on each cohort can be found in Supplementary Materials description of datasets

*DeKAF* Deterioration of kidney allograft function, *FRCBS* Finnish red cross blood service, *KiT-GENIE* kidney transplantation-genomic investigation of essential clinical concerns, *TL* transplant lines, *QUB* Queen’s University Belfast, *UKIRTC* United Kingdom and Ireland Renal Transplant consortium. *PRD* primary renal disease at transplant, *COD* cause of death, *PKD* polycystic kidney disease

The data suggest a significant difference in recipient graft survival times depending on the donor type. Organs from living donors have the longest (death-censored) graft survival, followed by those who died from other causes, with organs from deceased donors who died of stroke having the worst graft survival (*p* < 0.0001, see Supplementary Figure S1). Grafts from younger donors (< 45 years) also have better eGFR at 1-year post-transplant (see Supplementary Figure S2).

### Comparison of polygenic burden for stroke, intracranial aneurysm, and hypertension across donors and controls

**Fig. 1:**
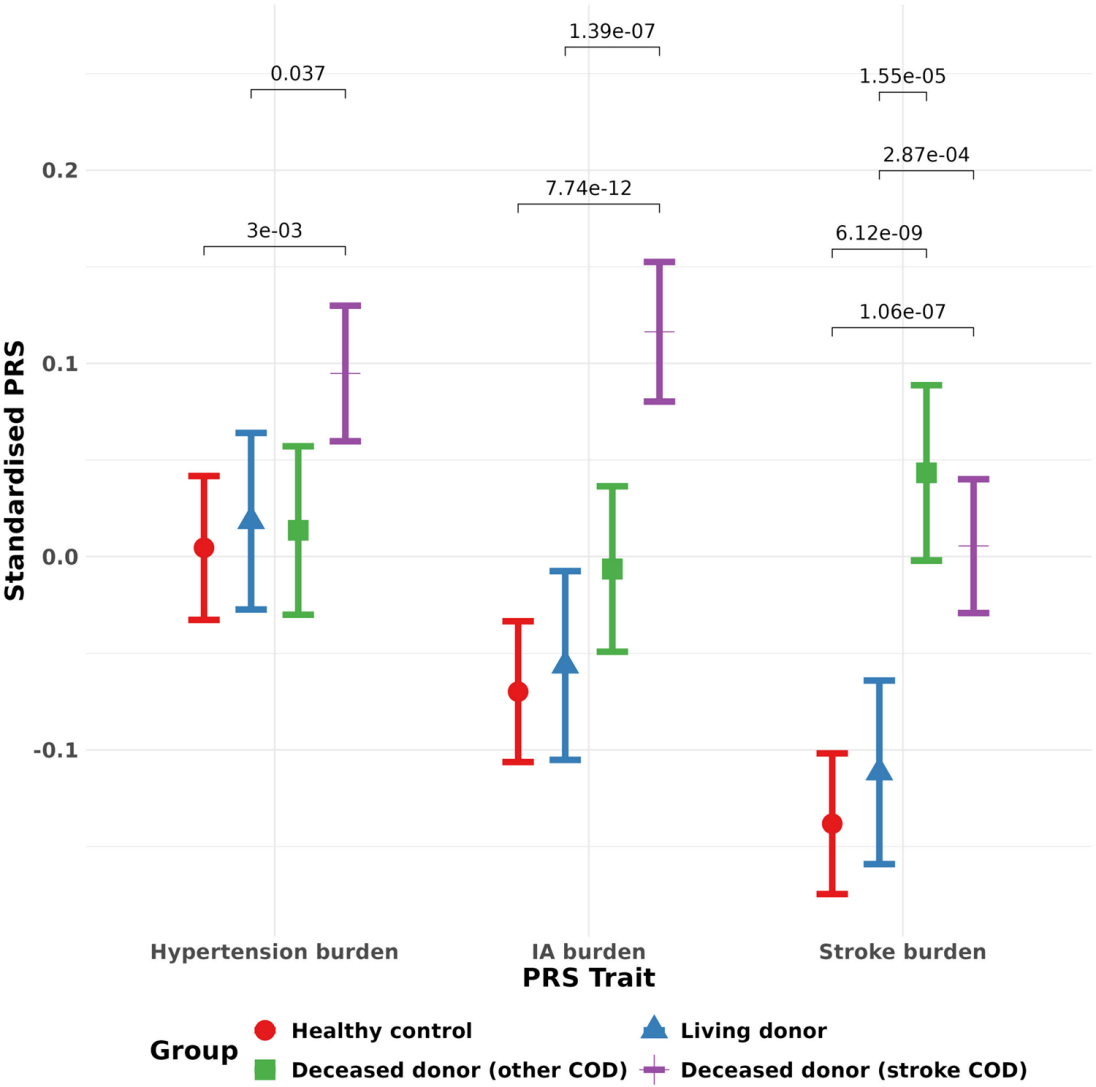
Cerebrovascular polygenic burden across donor type and controls. Mean value for each cerebrovascular polygenic risk score is given (along with 95% CI) for each donor type and the healthy controls. Bonferroni-adjusted *p*-value significance levels comparing the mean values in each group are above the error bars. Living donors have similar levels of polygenic burden to healthy controls. Deceased donors who died of stroke have elevated polygenic burden for each trait compared to healthy controls. Deceased donors who died of other causes appear to have slightly higher polygenic burden for each trait compared to healthy controls, but still lower than donors who died of stroke

We first tested the hypothesis that deceased donors have elevated genetic burden for the traits of intracranial aneurysm, stroke, and hypertension compared to both living donors and healthy controls. The donors deceased from stroke had significantly higher polygenic burden than the living donors (Bonferroni adjusted *p* values of 3e− 3, 7e− 12, 1e− 7 for hypertension, intracranial aneurysm, and stroke, respectively) (Fig. 1). As might be expected, healthy controls and living donors had a similar polygenic burden with no statistically significant difference for any of our traits of interest.

### Influence of polygenic burden for hypertension, intracranial aneurysm, and stroke on donor age of death

Next, we investigated if donor polygenic burden for hypertension, intracranial aneurysm, and stroke had an impact on donor age of death among the donors who died of stroke. We observed that a 1-standard deviation (SD) increase in intracranial aneurysm polygenic risk score results in a 0.57 year (~ 7 month) decrease in the age of death (95% CI 1.04–0.11 years, Supplementary figure S3).

### Donor polygenic burden and impact on graft outcome and function

**Fig. 2:**
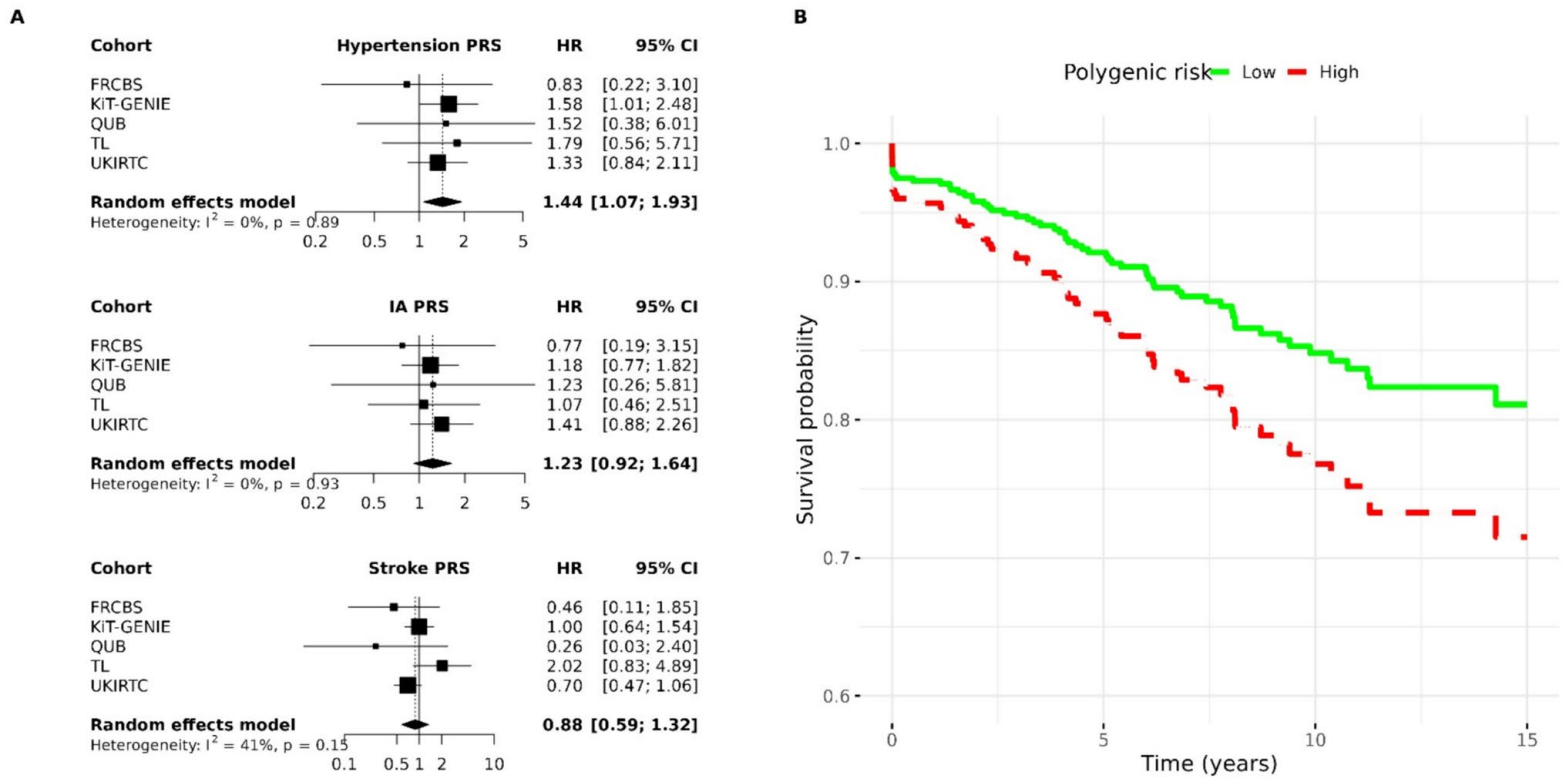
The influence of donor polygenic risk score on recipient graft survival. **A** Cox proportional hazards models for recipient graft survival (one model for each PRS: hypertension, IA, and stroke). Donor sex, donor age, recipient sex, recipient age, whether it was the recipient’s first transplant, year of transplant, and first 4 principal components of genetic ancestry are taken as covariates in each model. An individual who received a donor kidney in the top decile of polygenic risk for hypertension is 44% more likely to develop graft failure than those in the bottom decile of polygenic risk (95% CI 7%–93%). **B **Kaplan–Meier plot for graft survival split by donor hypertension polygenic risk (high vs low). *p*-value for differences in survival = 0.05. Finnish Red Cross Blood Service (FRCBS), kidney transplantation-genomic investigation of essential clinical concerns (KiT-GENIE), transplant lines (TL), United Kingdom and Ireland Renal Transplant consortium (UKIRTC)

Discretizing polygenic burden into 3 groups, (bottom 10%, middle 80% and top 10%, see Methods), we found that those with the highest hypertension polygenic burden were 44% more likely to develop graft failure compared to those with the lowest polygenic burden (95% CI 7%–93%) (Fig. 2A). We found no significant effect of intracranial aneurysm and stroke polygenic risk score on graft survival (Fig. 2A).

**Fig. 3:**
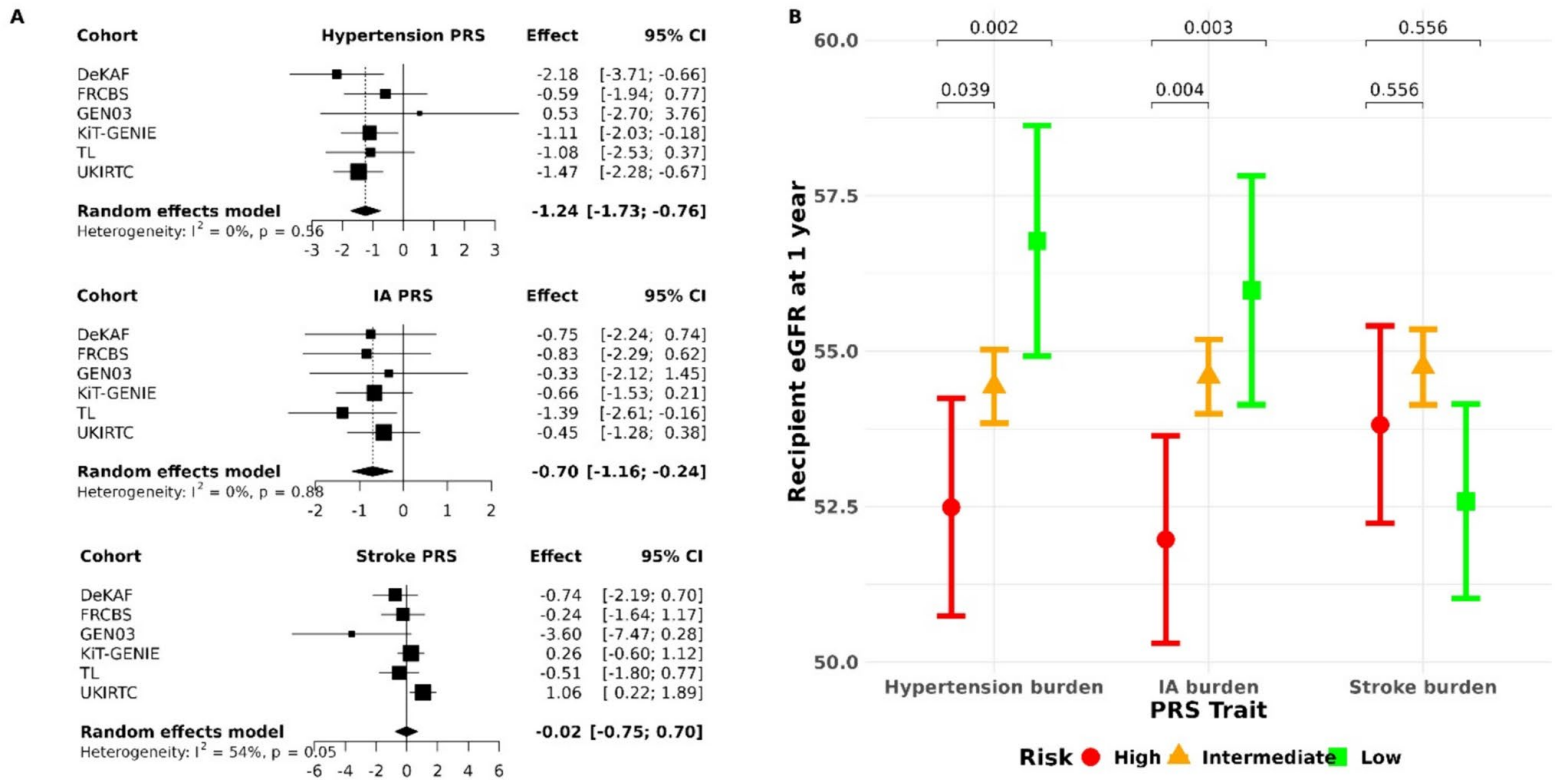
The influence of donor polygenic risk score on recipient eGFR at 1-year post-transplant. **A** Linear models for recipient eGFR at 1-year post-transplant (one model for each PRS: hypertension, IA, and stroke). Donor sex, donor age, recipient sex, recipient age, whether it was the recipient’s first transplant, year of transplant, and first 4 principal components of genetic ancestry are included as covariates in each model. A standard deviation increase in donor IA and hypertension polygenic risk score results in 0.7 and 1.24 mL/min/1.73 m^2^ decrease in recipient eGFR at 1-year post-transplant, respectively. eGFR was calculated using creatinine-based CKD-EPI equation. **B** Donor polygenic risk was split into low (bottom 10% of risk), intermediate (middle 80%) and high (top 10%). *p*-values are adjusted for multiple testing using a Bonferroni correction. Donor kidneys with low polygenic burden for hypertension and IA result in an approximately 5 mL/min/1.73 m^2^ higher eGFR at 1 year post-transplant

To investigate whether donor polygenic burden had an impact on recipient graft function we created a multivariate mixed effects linear model to predict eGFR at 1-year post-transplant based on donor polygenic risk score. We found that both hypertension and intracranial aneurysm polygenic risk scores were significant predictors of graft function, with estimated effect sizes of − 1.24 (95% CI − 1.73 to − 0.76) and − 0.70 (95% CI − 1.16 to − 0.24) (Fig. 3A). A one standard deviation increase in hypertension polygenic risk score thus resulted in a 1.24 mL/min/1.73 m^2^ decrease in eGFR at 1-year post-transplant.

We found significant differences in recipient eGFR at 1-year post-transplant between the individuals with low, intermediate and high polygenic burden for the traits of intracranial aneurysm and hypertension (Fig. 3B). Of interest, we found that the value for eGFR in the individuals with high burden for hypertension was 52.5 mL/min/1.73 m^2^, whereas in the low burden individuals it was 57.1 mL/min/1.73 m^2^ (Bonferroni adjusted *p*-value: 0.002). The corresponding values for intracranial aneurysm were 52.0 mL/min/1.73 m^2^ and 56.0 mL/min/1.73 m^2^ (Bonferroni adjusted *p*-value: 0.003).

We analysed the impact of extremes of polygenic risk score for intracranial aneurysm, hypertension and stroke on eGFR at 1 year. Comparing different definitions of high and low polygenic risk score we have shown the most marked effect on eGFR when individuals in the top 10% for polygenic risk score for hypertension were compared with those in the bottom 10%. These groups showed a difference of 4.3 mL/min/1.73 m^2^ in GFR at 1-year post-transplant. Similar observations were made for other groups (Supplementary Table S3).

Similarly, we created a multivariate mixed effects linear model to predict recipient eGFR at 5-years post-transplant based on donor polygenic risk score for hypertension, stroke, and intracranial aneurysm but did not find any impact at 5 years, possibly due to a lack of power, resulting from much more missing data in relation to eGFR at 5 years post-transplant.

## Discussion

Here we investigated the impact of donor polygenic burden for cerebrovascular traits on donor age of death and recipient graft outcomes, in 6666 European ancestry kidney transplants. We found that kidney donors who died of stroke had elevated intracranial aneurysm and hypertension polygenic risk scores compared to healthy controls and living donors. The same two polygenic risk scores also had a significant impact on donor age of death and recipient graft function. To our knowledge, this is the first time that polygenic burden for traits related to cerebrovascular disease have been explored in transplant donors.

We have observed a significant effect of high polygenic risk score for hypertension having reduced graft survival when compared to those with low polygenic risk score for hypertension. This is also associated with significant association with eGFR at 1 year with patients in the highest decile for hypertension polygenic risk score having a 4.6 mL/min/1.73 m^2^ lower eGFR than those with the lowest decile. Similarly, the results for intracranial aneurysm demonstrated a 4 mL/min/1.73 m^2^ difference between the highest and lowest decile. The observed association between donor polygenic risk score for hypertension and eGFR is notable considering the recent demonstration of lower eGFR in kidneys from hypertensive living donors [23].

This work has several limitations. The predictive ability of any polygenic risk score depends on the power of the genome-wide association studies used to generate it. While we leveraged the largest intracranial aneurysm, stroke and hypertension genome-wide association studies available, future genome-wide association studies for these traits will only increase in size, enabling more powerful polygenic risk scores. Focused genome-wide association studies for subarachnoid haemorrhage resulting in the death of the patient would enable more targeted donor polygenic risk scores for use in the transplant setting.

Just over 15% (*n* = 1047) of transplants in our study date from before the millennium, providing both advantages and disadvantages. Whilst we have decades of follow-up for some transplants, outcomes have improved significantly as the field has evolved. We have attempted to control for this by including the year of transplant as a covariate. However, our analysis also lacked some known predictors of transplant outcome, including the kidney donor profile index (KDPI) and expanded criteria donor (ECD). As most of the transplants predated the introduction of the kidney donor profile index and expanded criteria donor, we were unable to control for these. As a result, it was impossible to compare the features used in the clinic to guide donor eligibility. However, donor sex was included as a covariate, as were donor age and donor cause of death, two of the most important criteria in calculating the kidney donor profile index. Data points for eGFR at 5 years post-transplant were also unavailable in 58% of the cohort.

Immunological mechanisms are considered a major contributor to graft loss. Our results are independent of immunological mechanisms and demonstrate that donor genetic mechanisms associated with hypertension and arterial disease influence long-term graft function. These observations may explain some of the mechanisms associated with worse long-term function from deceased donors, particularly intracranial haemorrhage donors, compared to live donors, and this strategy may be applicable to all types of transplanted organs.

With genome-wide association studies becoming increasingly powerful and more predictive, in the future polygenic risk scores may be able to explain more of the heterogeneity in graft survival and function post-transplant, and thus play a role in transplant allocation decisions. Those who receive a kidney with a high polygenic risk score for hypertension, for instance, may need more aggressive post-transplant monitoring.

Donors who die of cerebrovascular disease likely have a generalized pan-vascular disease process [24] which is reflected in the increased polygenic risk score for hypertension. These polygenic risk score signals may represent diverse physiological mechanisms that likely affect the transplanted kidney and contribute to the reduced graft function in kidneys that come from donors with high polygenic burden. As polygenic scores become more powerful, donor polygenic risk scores could potentially be included in transplant allocation decisions in the future, but more research is required in this area, particularly in terms of the interaction between donor and recipient polygenic risk scores.

Collectively, the results presented here demonstrate the impact of inherited factors associated with donors' death on long-term graft survival and function. Collaborative efforts on cohorts with long and detailed clinical follow-up including all the variables utilized within the kidney donor profile index system are required to validate these findings before they can have a role in future organ allocation policies.

## Supplementary Information

Below is the link to the electronic supplementary material.

Supplementary file1 (DOCX 1068 KB)

## Data Availability

The data used in this study are not publicly available due to concerns regarding patient confidentiality and privacy. Access to the data can be requested from each participating center individually, subject to their respective data sharing policies and ethical considerations.

## Abbreviations

CI: Confidence interval
DeKAF: Deterioration of kidney allograft function
eGFR: Estimated glomerular filtration rate
FRCBS: Finnish red cross blood service
GWAS: Genome-wide association study
HLA: Human leukocyte antigen
IA: Intracranial aneurysm
IS: Ischaemic stroke
KiT-GENIE: Kidney transplantation-genomic investigation of essential clinical concerns
PRS: Polygenic risk score
QUB: Queen’s University Belfast
SD: Standard deviation
SNP: Single nucleotide polymorphism
TL: Transplant lines
UKIRTC: United Kingdom and Ireland Renal Transplant consortium
