# Supplementary material for: Donor genetic burden for cerebrovascular risk and kidney transplant outcome
Source: J Nephrol. 2024 May 29;37(6):1643–52. doi: 10.1007/s40620-024-01973-0 (PMC11473625; doi:10.1007/s40620-024-01973-0)
Supplement: Supplementary file 1 — Supplementary file1 (DOCX 1068 KB) [file 40620_2024_1973_MOESM1_ESM.docx]

Contents

[Data characteristics 3](#_Toc164413710)

[Description of cohorts 3](#_Toc164413711)

[Supplementary table S1: Summary statistics for the meta-analysis split by donor type. 5](#_Toc164413712)

[Supplementary figure S1: The influence of donor type on graft survival. 6](#_Toc164413713)

[Supplementary figure S2: The influence of donor age on recipient eGFR at 1-year post-transplant. 7](#_Toc164413714)

[Data QC and processing 8](#_Toc164413715)

[Studies used to generate PRS 9](#_Toc164413716)

[Supplementary table S2: Details of studies used to generate PRS 9](#_Toc164413717)

[Supplementary figure S3: The influence of donor PRS on donor age of death among donors who died of stroke 10](#_Toc164413718)

[Supplementary figure S4: Testing the assumptions of the linear model for post-transplant eGFR (at 1 year) 11](#_Toc164413719)

[12](#_Toc164413720)

[Supplementary figure S5: Testing the assumptions of the linear model for post-transplant eGFR (at 5 years) 12](#_Toc164413721)

[Supplementary table S3: Recipient eGFR at 1 and 5 years’ post-transplant stratified by donor polygenic burden 13](#_Toc164413722)

[Bibliography 14](#_Toc164413723)

# Data characteristics

## Description of cohorts

We assembled 7 different cohorts for this study, all of European ancestry:

**United Kingdom and Ireland Renal Transplant consortium (UKIRTC):** 2104 genotyped deceased donor transplants from all kidney transplant centres in the UK and Ireland that took place between 1987 and 2007^1^.

**Transplant Lines (TL):** 1169 genotyped donors (262 living, 907 deceased) from the Netherlands. This cohort is a single-centre biobank from the University Medical Centre Groningen including all different types of solid organ transplant recipients and living organ donors^2^. These individuals were imputed using the 1000 Genomes imputation panel.

**Deterioration of Kidney Allograft Function (DeKAF) and GEN03:** The Deterioration of Kidney Allograft Function (DeKAF) and GEN03 studies are cohorts of transplant recipients from 7 transplant centres in the US and Canada^3,4^. They contributed 684 and 476 donor-recipient pairs, respectively, to this study.

**Kidney Transplantation - Genomic Investigation of Essential clinical concerns (KiT-GENIE):** 1858 donors (319 living, 1539 deceased) were collected from the French monocentric KiT-GENIE cohort^5^.

**Finnish Red Cross Blood Service (FRCBS):** 929 genotyped deceased donor pairs transplanted in a single centre in Helsinki between 2007 and 2017^6^.

**Queen’s University Belfast (QUB):** 133 genotyped deceased donor-recipient transplant pairs from across Northern Ireland. These samples were distinct from the samples included in the UKIRTC^7^.

Unless otherwise stated, the autosomal genotypes of donors and controls were imputed using either the Haplotype Reference Consortium r1.1 2016 on the Sanger imputation server, with phasing using Eaglev2.4. All data was then filtered for standard QC parameters (supplementary materials section on Data QC and processing).

| **Variable** | **Overall** | **Living** | **Other COD** | **Stroke COD** |
| --- | --- | --- | --- | --- |
| **Number of patients** | 6,666 | 1,582 | 1,971 | 3,113 |
| **Donor age (years), mean (range)** | 49 (18 - 90) | 45 (18 - 73) | 44 (18 - 90) | 54 (18 - 89) |
| **Female donor, n (%)** | 3106 (47) | 939 (59) | 566 (29) | 1601 (51) |
| **Primary renal disease, n (%)** |  |  |  |  |
| Glomerulonephritis | 772 (12) | 352 (22) | 161 (8) | 259 (8) |
| IgA nephropathy | 313 (5) | 16 (1) | 107 (5) | 190 (6) |
| Other | 3283 (49) | 622 (39) | 1074 (54) | 1587 (51) |
| Polycystic kidney disease | 990 (15) | 256 (16) | 270 (14) | 464 (15) |
| Type II diabetes | 366 (6) | 261 (16) | 32 (2) | 73 (2) |
| Unknown | 942 (14) | 75 (5) | 327 (17) | 540 (17) |
| **First transplant, n (%)** | 5852 (88) | 1402 (89) | 1714 (87) | 2736 (88) |
| **Year of transplant, mean (range)** | 2007 (1981 - 2020) | 2010 (1993 - 2020) | 2005 (1981 - 2019) | 2006 (1986 - 2019) |
| **Follow up (years), mean (range)** | 6 (0 - 25) | 3 (0 - 20) | 7 (0 - 25) | 7 (0 - 24) |
| **Graft status, n (%)** |  |  |  |  |
| Censored | 5577 (84) | 1528 (97) | 1603 (81) | 2446 (79) |
| Rejected | 1089 (16) | 54 (3) | 368 (19) | 667 (21) |
| **eGFR at 1 year (mL/min/1.73m^2^), mean (range)** | 54 (4 - 185) | 62 (4 - 185) | 56 (4 - 135) | 48 (5 - 124) |
| Unknown, n | 1030 | 21 | 339 | 670 |
| **eGFR at 5 year (mL/min/1.73m^2^), mean (range)** | 51 (3 - 124) | 60 (5 - 113) | 55 (3 - 124) | 48 (4 - 121) |
| Unknown, n | 3865 | 1332 | 960 | 1573 |

## Supplementary table S1: Summary statistics for the meta-analysis split by donor type

*First transplant refers to whether it is the recipient’s first transplant or not. The numbers in each cohort refer to the number of donor kidneys rather than just the number of donors, as one deceased donor may donate two kidneys.*


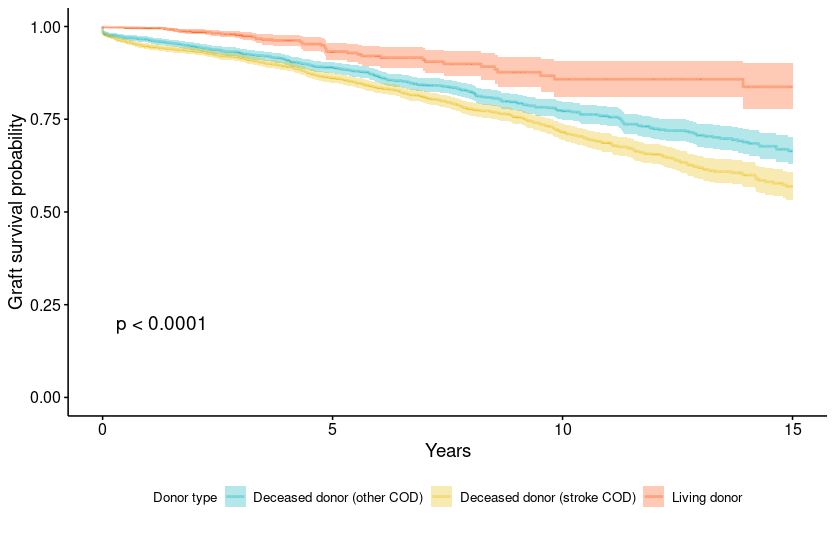


## Supplementary figure S1: The influence of donor type on graft survival

*Grafts that come from living donors have the best survival, followed by grafts from deceased donors who died of other causes of death, with grafts from deceased donors who died of stroke having the worst outcomes.*

***
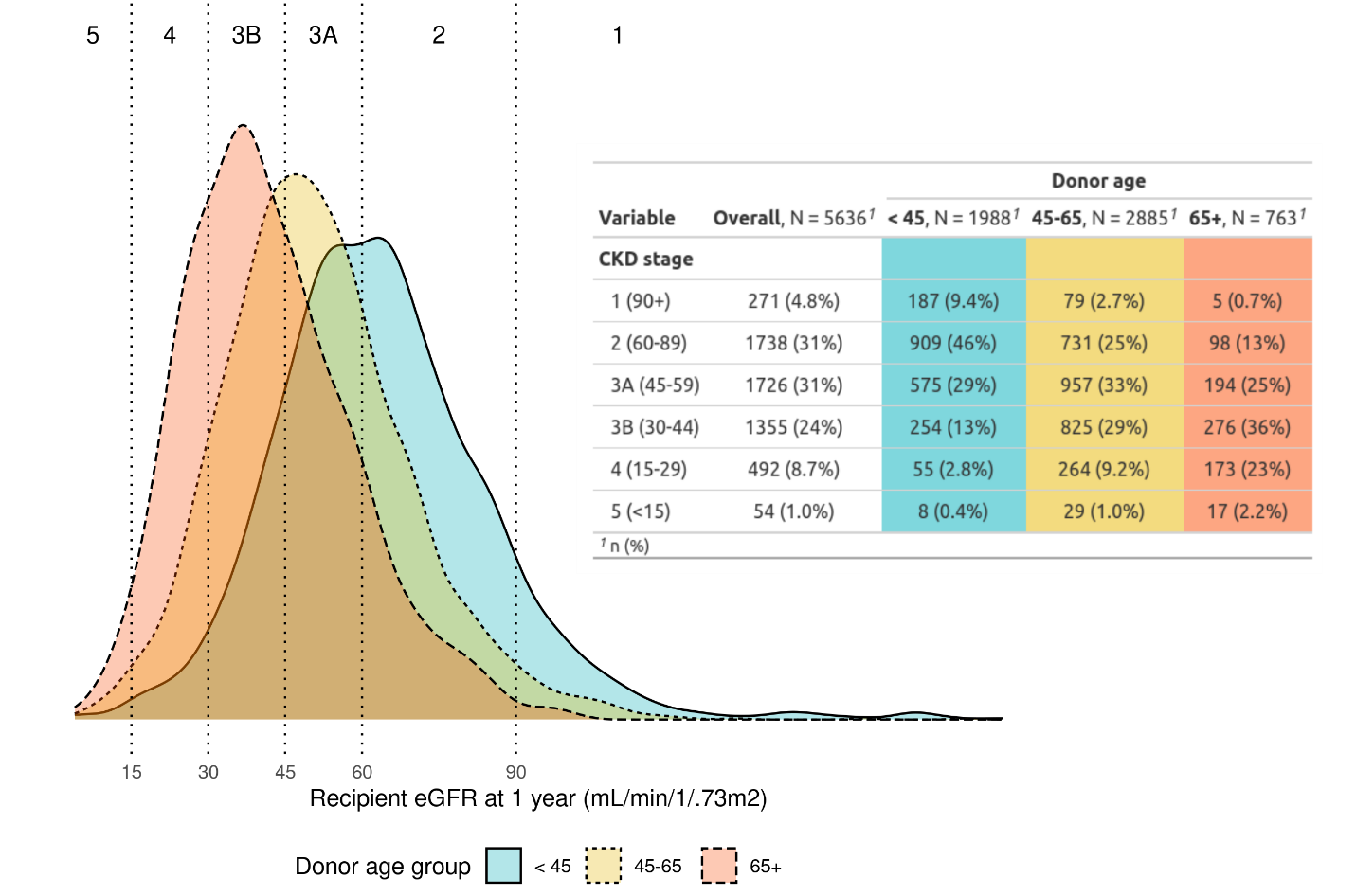
***

## Supplementary figure S2: The influence of donor age on recipient eGFR at 1-year post-transplant

*Grafts that come from younger donors have the best eGFR at 1-year post-transplant.*

# Data QC and processing

The following criteria were used to determine inclusion in this study:

1. Standard QC parameters of minor allele frequency of 0.02, missingness 0.05, genotyping rate 0.05.
2. All participants were unrelated to the level of 3^rd^ degree. This was determined using KING^8^.
3. All participants must be of European ancestry. This was determined using principal components analysis (PCA) with the reference European ancestry population from 1000 Genomes.

# Studies used to generate PRS

| **GWAS Trait** | **Study** | **Sample size** | **# Loci** | **# SNPs used (after pruning)** | **SNP based heritability** |
| --- | --- | --- | --- | --- | --- |
| Intracranial aneurysm (IA) | Bakker et al. 2020^9^ | 7,495 cases and 71,934 controls | 17 | 50,377 | 21.6% |
| Hypertension | Wenjian et al. 2020^10^ | 76,566 cases and 206,305 controls | 204 | 63,640 |  |
| Stroke | Malik et al. 2018^11^ | 67,162 cases and 454,450 controls | 32 | 65,266 |  |

## Supplementary table S2: Details of studies used to generate PRS

*Sample sizes and reference study for each trait for which a PRS was generated. # Loci is the number of genome-wide significant loci identified in the original study. # SNPs used refers to the number of Single Nucleotide Polymorphisms (SNPs) used by PRSice in calculating the PRSs after pruning and thresholding.*


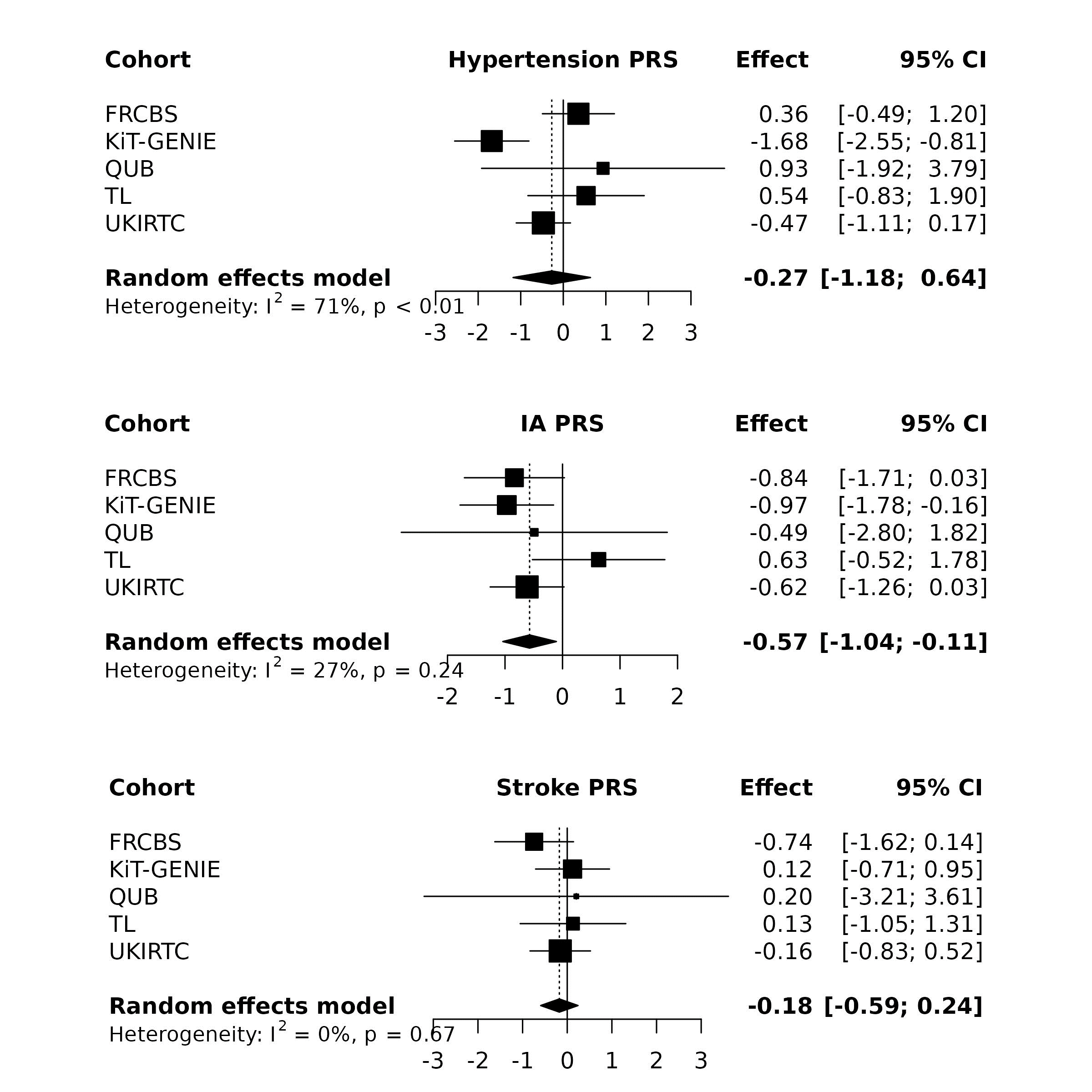


## Supplementary figure S3: The influence of donor PRS on donor age of death among donors who died of stroke

Linear models for donor age of death in deceased donors who died of stroke (one model for each PRS: hypertension, IA, and stroke). Donor sex and first 4 principal components of genetic ancestry are included as covariates in each model. A standard deviation increase in donor IA PRS results in a 0.57 year earlier donor age of death (95% CI: -1.04—0.11 years). Stroke and hypertension PRS do not have a significant impact on donor age of death. Further details of each cohort are given in the supplementary materials.


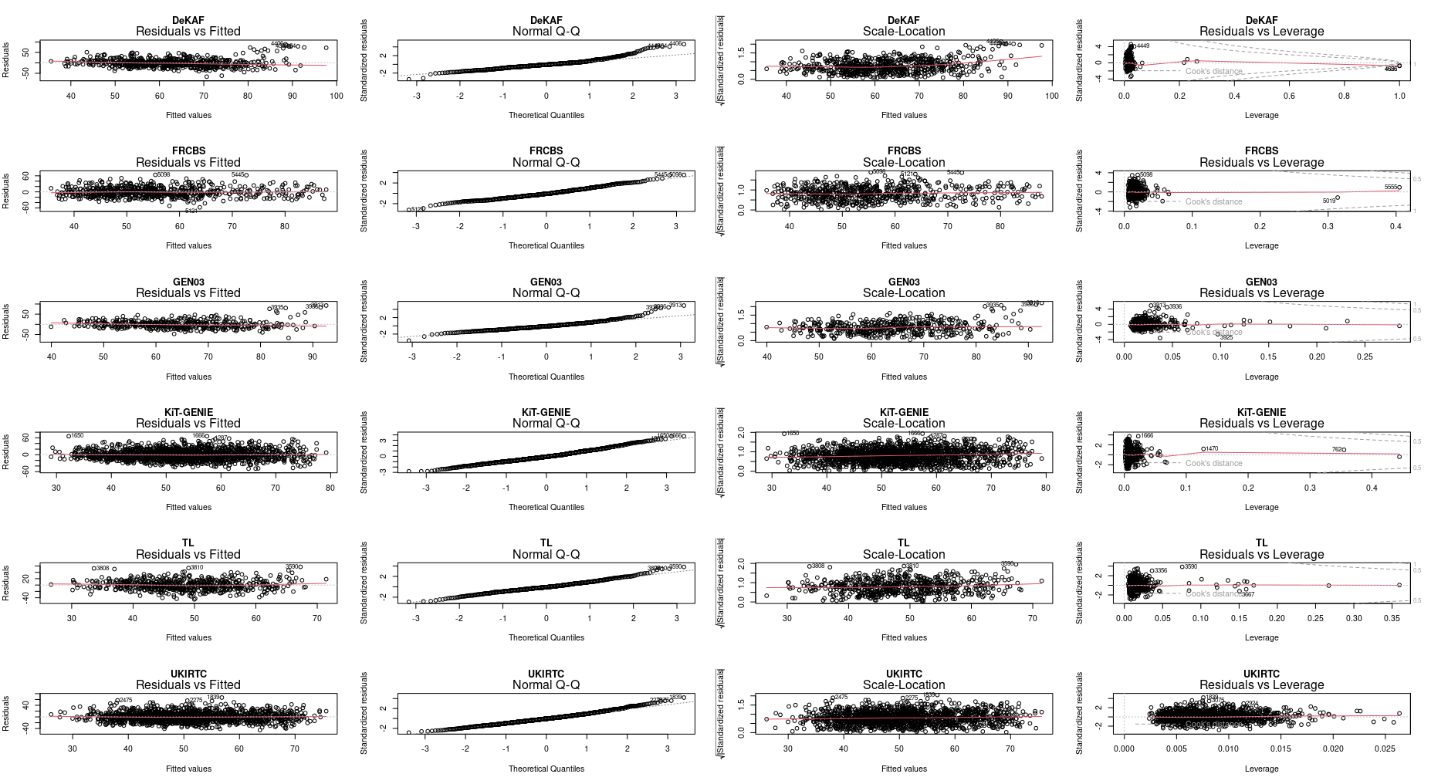


## Supplementary figure S4: Testing the assumptions of the linear model for post-transplant eGFR (at 1 year)

*Testing the assumptions of the linear model to predict recipient eGFR at 1-year post-transplant. The following assumptions were tested: non-linearity, homogeneity of variance, influential observations, collinearity, normality of residuals, and normality of random effects. All the assumptions are satisfied.*

##
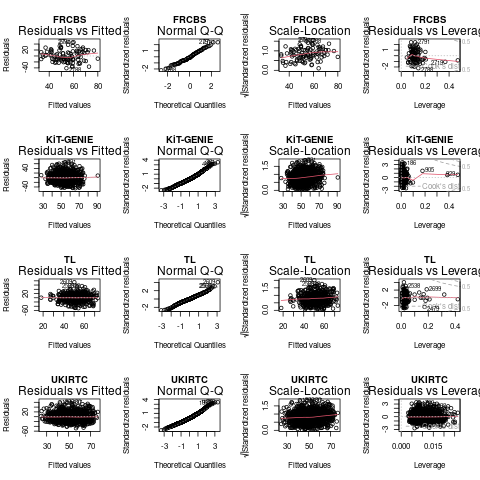


## Supplementary figure S5: Testing the assumptions of the linear model for post-transplant eGFR (at 5 years)

*Testing the assumptions of the linear model to predict recipient eGFR at 5-years post-transplant. The following assumptions were tested: non-linearity, homogeneity of variance, influential observations, collinearity, normality of residuals, and normality of random effects. All the assumptions are satisfied for each cohort.*

|  |  | **eGFR at 1 year post-transplant** | | | | | **eGFR at 5 years post-transplant** | | | | |
| --- | --- | --- | --- | --- | --- | --- | --- | --- | --- | --- | --- |
| **PRS** | **Threshold** | **High burden** | **Low burden** | **Difference** | **p** | **Adjusted p** | **High burden** | **Low burden** | **Difference** | **p** | **Adjusted p** |
| **Hypertension** | Top 2% vs bottom 2% | 54.3 | 56.7 | 2.4 | 0.469 | 1 | 49.8 | 53.2 | 3.4 | 0.463 | 1 |
|  | **Top 5% vs bottom 5%** | **53.2** | **58** | **4.8** | **0.016** | **0.064** | 52 | 54.4 | 2.4 | 0.38 | 1 |
|  | **Top 10% vs bottom 10%** | **52.5** | **57.1** | **4.6** | **0.001** | **0.004** | 50.6 | 54 | 3.4 | 0.053 | 0.212 |
|  | **Top 20% vs bottom 20%** | **52.3** | **55.8** | **3.5** | **<0.001** | **<0.001** | 50.9 | 52.8 | 1.9 | 0.139 | 0.556 |
| **IA** | Top 2% vs bottom 2% | 51 | 52.9 | 1.9 | 0.517 | 1 | 49 | 47 | -2 | 0.634 | 1 |
|  | Top 5% vs bottom 5% | 51.8 | 54.2 | 2.4 | 0.2 | 0.8 | 49 | 52 | 3 | 0.254 | 1 |
|  | **Top 10% vs bottom 10%** | **52** | **56** | **4** | **0.002** | **0.008** | 49.4 | 53.2 | 3.8 | 0.028 | 0.112 |
|  | **Top 20% vs bottom 20%** | **52.5** | **55.7** | **3.2** | **<0.001** | **<0.001** | 50.7 | 52.8 | 2.1 | 0.078 | 0.312 |
| **Stroke** | Top 2% vs bottom 2% | 56 | 50.2 | -5.8 | 0.038 | 0.152 | 51.9 | 48.3 | -3.6 | 0.358 | 1 |
|  | Top 5% vs bottom 5% | 54.2 | 52.5 | -1.7 | 0.314 | 1 | 51.2 | 49.7 | -1.5 | 0.517 | 1 |
|  | Top 10% vs bottom 10% | 53.8 | 52.6 | -1.2 | 0.278 | 1 | 50 | 51 | 1 | 0.532 | 1 |
|  | Top 20% vs bottom 20% | 53.8 | 54.2 | 0.4 | 0.646 | 1 | 51.7 | 50.7 | -1 | 0.42 | 1 |

## Supplementary table S3: Recipient eGFR at 1 and 5 years’ post-transplant stratified by donor polygenic burden

eGFR is in mL/min/1.73m^2^. P-values are adjusted for multiple testing. We see that there is a difference of between 2.4 and 4.8 mL/min/1.73m^2^ between the low and high hypertension burden donor kidneys and a difference ranging between 1.9 and 4.0 mL/min/1.73m^2^ for IA burden donor kidneys. There is no significant difference for stroke. Statistically significant differences are highlighted in bold.

# Bibliography

1. Hernandez-Fuentes MP, Franklin C, Rebollo-Mesa I, et al. Long- and short-term outcomes in renal allografts with deceased donors: A large recipient and donor genome-wide association study. *Am J Transplant*. 2018;18(6):1370-1379. doi:10.1111/ajt.14594

2. Eisenga MF, Gomes-Neto AW, Van Londen M, et al. Rationale and design of TransplantLines: A prospective cohort study and biobank of solid organ transplant recipients. *BMJ Open*. 2018;8(12):1-13. doi:10.1136/bmjopen-2018-024502

3. Matas AJ, Fieberg A, Mannon RB, et al. Long-term follow-up of the DeKAF cross-sectional cohort study. *Am J Transplant*. 2019;19(5):1432-1443. doi:10.1111/ajt.15204

4. Mohamed ME, Schladt DP, Guan W, et al. Tacrolimus Troughs and Genetic Determinants of Metabolism in Kidney Transplant Recipients: A comparison of four ancestry groups. *Am J Transplant*. 2019;19(10):2795-2804. doi:10.1111/ajt.15385

5. Garrigue V, Szwarc I, Giral M, et al. Influence of anemia on patient and graft survival after renal transplantation: Results from the French DIVAT Cohort. *Transplantation*. 2014;97(2):168-175. doi:10.1097/TP.0b013e3182a94a4d

6. Markkinen S, Helanterä I, Lauronen J, Lempinen M, Partanen J, Hyvärinen K. Mismatches in Gene Deletions and Kidney-related Proteins as Candidates for Histocompatibility Factors in Kidney Transplantation. *Kidney Int Reports*. 2022;7(11):2484-2494. doi:10.1016/j.ekir.2022.08.032

7. Steers NJ, Li Y, Drace Z, et al. Genomic Mismatch at LIMS1 Locus and Kidney Allograft Rejection . *N Engl J Med*. 2019;380(20):1918-1928. doi:10.1056/nejmoa1803731

8. Manichaikul A, Mychaleckyj JC, Rich SS, Daly K, Sale M, Chen WM. Robust relationship inference in genome-wide association studies. *Bioinformatics*. 2010;26(22):2867-2873. doi:10.1093/bioinformatics/btq559

9. Bakker MK, van der Spek RAA, van Rheenen W, et al. Genome-wide association study of intracranial aneurysms identifies 17 risk loci and genetic overlap with clinical risk factors. *Nat Genet*. 2020;52(12):1303-1313. doi:10.1038/s41588-020-00725-7

10. Bi W, Fritsche LG, Mukherjee B, Kim S, Lee S. A Fast and Accurate Method for Genome-Wide Time-to-Event Data Analysis and Its Application to UK Biobank. *J Clean Prod*. 2020;107(2):222-233. doi:10.1016/j.ajhg.2020.06.003

11. Malik R, Chauhan G, Dichgans M. Multiancestry genome-wide association study of 520,000 subjects identifies 32 loci associated with stroke and stroke subtypes. *Nat Genet*. 2018;50(12):524–537. doi:10.1038/s41588-018-0058-3
